# Thermal performance of a moving fin with temperature-dependent thermal conductivity in convective and radiative environment

**DOI:** 10.1016/j.heliyon.2025.e42329

**Published:** 2025-01-29

**Authors:** Yanqun Han, Xulong Peng

**Affiliations:** a School of Civil Engineering, Central South University, Changsha 410075, Hunan, PR China; b School of Civil Engineering, Changsha University of Science and Technology, Changsha 410114, Hunan, PR China

**Keywords:** Approximate temperature, Convective-radiative fin, Temperature-dependent thermal conductivity, Fin efficiency, Nonlinear heat transfer problem

## Abstract

This article studies the thermal performance of a moving fin with temperature-dependent thermal conductivity in a convective and radiative environment. It corresponds to a nonlinear heat transfer problem related to the nonlinear ordinary differential equation (NODE) for the unknown temperature excess. The NODE is solved by converting it to a nonlinear Fredholm integral equation. An approximate temperature distribution is determined in the quadratic form for arbitrary values of the Biot and Peclet numbers. A comparison of our results with the previous ones indicates satisfactory accuracy of the obtained solution. The fin efficiency is also given explicitly in terms of prescribed parameters and calculated numerically. The heat dissipation to the surrounding medium due to convection and radiation is analyzed for various speeds of a moving fin. The influences of thermal conductivity, heat convection, radiation, and moving speed of the fin on the temperature distribution and thermal performance are elucidated.

## Nomenclature

A: cross-sectional area m^2^
Bi: Biot number
h: convection coefficient W/m^2^K
k(T): thermal conductivity depending on temperature W/mK
ka: thermal conductivity at temperature $T_{a}$ W/mK
L: fin length m
P: cross-sectional perimeter m
Pe: Peclet number
$Q_{actual}/Q_{ideal}$: actual/ideal heat losses W
Qc: convection loss of heat W
Qr: radiation loss of heat W
*q*: heat transfer rate W
T: temperature of fin at any location K
Ta: convection sink temperature K
Tb: temperature at base K
Ts: radiation sink temperature K
X: distance from the fin's tip m

**Greek symbols**
α: exponential index of thermal conductivity
ε: surface emissivity
λ: parameter to describe thermal conductivity variation
η: fin efficiency
σ: Stefan-Boltzmann constant W/m^2^K^4^
θ: temperature excess

## 1 Introduction

Fins have been widely used for cooling electric transformers, the cylinders of aircraft engines, air conditioning, refrigeration, and other cooling elements for computer equipment. The rate of heat transfer can be enhanced if an optimal shape and proper material are selected. The thermal performance of fins depends on both convection transfer and radiation transfer when heat conducts from the root or base to the fins. Fins may be used to fabricate desirable shapes to improve the thermal performance of heat transfer equipments in engineering fields, in particular for those with an uneasy increase in the heat transfer coefficient [1].

For those fins with low convection heat transfer coefficients, the heat dissipation from the surface due to radiation has as much as the heat dissipation due to convection. Consequently, it is necessary to consider radiation and convection simultaneously in assessing thermal high-performances of fins with low convection coefficients. Considering three typical means of heat transfer in fins, considerable work is devoted to the thermal performance analysis of fins with various shapes [2], [3], [4], including convection heat loss and/or radiation heat loss. If the temperature variation in fins is relatively large, the thermal conductivity, convection heat coefficient, and even radiation heat coefficient are no longer considered constant, but depend on the varying temperature. For nonlinear fin problems related to convective and radiative heat transfer, a lot of numerical approaches applied to convective-conductive heat transfer or radiative-conductive heat transfer have been developed to handle the temperature change of convective-radiative-conductive fins, including the Adomian's decomposition method [5], [6], the differential transformation method [7], [8], the Runge-Kutta method [9], [10], the collocation method [11], [12], the homotopy perturbation method [13], [14], the integral equation method [15], [16], the series method [17], [18], [19], and other analytic methods for exact closed-form solution in implicit form [20], [21], [22].

On the other hand, in processes like extrusion, glass fiber drawing, and hot rolling and casting, in order to cool the material to a desirable temperature before it is spooled or removed, the material in a state of continuous motion is allowed to exchange heat with its ambient environment. The velocity of the material is also significant and plays a key role in affecting the temperature distribution due to its motion from a high-temperature furnace to a colder ambient. When considering nonlinear moving fin problems with convective-radiative heat loss, many researchers extended various analytical and numerical techniques to handle the temperature change along with the thermal performance of the fins. Singhal et al. [23] conducted an experimental and computational inverse thermal analysis of transient, nonlinear heat flux in circular pin fin with temperature-dependent thermal properties. In theoretical analyses, Aziz and Lopez [24] first proposed a numerical heat transfer analysis for a continuously moving sheet/rod of variable thermal conductivity with both convection and radiation losses by using a fourth-fifth order Runge-Kutta-Fehlberg method. Aziz and Khani [25] applied the homotopy analysis method to derive a series expression for the temperature in a moving convective-radiative fin of variable thermal conductivity. Torabi et al. [26] developed the differential transformation method to determine the temperature change of a moving convective and radiative fin having temperature-dependent thermal conductivity. Singla and Das applied the Adomian decomposition method to study a rectangular fin or a stepped fin with temperature-dependent nonlinear heat transfer and heat dissipation [27], [28], [29]. Singla and Das [30] employed the Adomian decomposition method to also obtain the temperature field and the fin efficiency for a conductive–convective and radiating moving fin having variable thermal conductivity. Bhanja et al. [31] adopted the Adomian decomposition method to establish an analytical approach for the determination of temperature excess and fin efficiency for a porous moving fin exposed in convective–radiative environment. Sun and Xu [32] studied the thermal performance of a continuously moving radiative-convective fin with complex cross-section and multiple nonlinearities. For moving fins having temperature-dependent thermal conductivity, heat transfer coefficient, and heat generation, Dogonchi and Ganji [33] addressed a nonlinear convection-radiation heat transfer problem. Ma et al. [34] employed the spectral element method to investigate combined conductive, convective, and radiative heat transfer for moving porous fins. Turkyilmazoglu [35] analyzed the exponential profile of moving fins on heat transfer exposed to heat generation or absorption. Shateri and Salahshour [36] formulated a study of comprehensive thermal performance of a convection-radiation longitudinal porous fin with various profiles including rectangular, trapezoidal, and concave cross-sections. Ndlovu and Moitsheki [37] utilized the variational iteration method to study heat transfer related to radial moving fins of temperature-dependent thermal conductivity and heat transfer coefficient. Buonomo et al. [38] adopted the Adomian decomposition method to analyze heat transfer of a rectangular porous fin in local thermal non-equilibrium model. Din et al. developed the differential transformation method and the Runge-Kutta method to deal with convective-radiative moving exponential porous fins with internal heat generation [39], [40]. For a wet porous inclined fin moving in a convective-radiative hybrid nanofluid or exposed to magnetic field, Pavithra and Gireesha employed the Adomian decomposition Sumudu transform method to give a tedious series solution of temperature [41], [42]. Based on the Runge-Kutta Fehlberg technique and finite element method, respectively, Gireesha et al. investigated the thermal behavior of a moving longitudinal porous fin with thermal radiation and natural convection condition [43], [44], [45]. Ullah et al. [46] applied the differential transformation method to determine the temperature change for a moving convective-radiative triangular porous fin with heat generation. Gamaoun et al. [47] addressed the temperature distribution of a moving longitudinal radiative-convective dovetail fin. Sowmya et al. [48] utilized the two-dimensional differential transform method with multivariate Pade approximation to analyze the transient temperature distribution in a convective-radiative moving fin.

Although there is a great amount of literature focusing on the analytical and numerical methods for determining the temperature distribution of a moving convective-radiative fin in various environment, the temperature change derived in literature is often in the series form (e.g. [25], [26], [31], [33], [40], [41], [42], [46], [48]) or in numerical results (e.g. [32], [34], [39], [43], [47]). However, these solutions have a noticeable shortcoming: they are either completely numerical or quite complicated. Owing to the difference of fin materials and variation of the heat environment, when some parameters vary, the computation of these solution needs to spend an amount of time. Moreover, due to the nature of numerical results, it is difficult to comprehensively understand the dependence of the temperature distribution on the specified physical quantity. It leads to that the temperature distribution of a moving fin is quite complicated, albeit in explicit form sometime like the series form with the aid of Adomian decomposition method and differentiation transformation method, etc. Therefore, it is still inconvenient in determining the temperature distribution. For example, for a nonlinear heat transfer problem related to a moving fin exposed in convective-radiative environment, an explicit expression for the temperature distribution dependent on the Biot and Peclet numbers is still lacking, to the best of our knowledge. For practical situations, an explicit analytical solution is much desired in a simple closed form, even in approximate form. Such solutions are particularly useful in design and optimization.

This article proposes an approximate integral equation method to study the thermal performance of a moving conductive fin exposed to convective-radiative environment. The heat dissipation to the surrounding medium due to convection and radiation is analyzed for various speeds of a moving fin. The thermal conductivity of the fin is assumed to be linearly dependent on the temperature. The main aim of this paper is to derive a closed-form approximate temperature distribution with satisfactory accuracy in simple quadratic form. The fin efficiency is evaluated. The impacts of the thermal conductivity, Biot and Peclet numbers on the temperature change and the fin efficiency are discussed and elucidated in graph.

## 2 Statement of the problem

**Figure 1:**
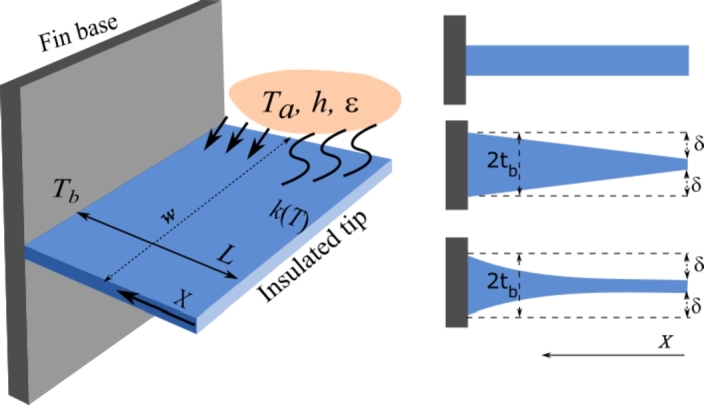
Schematic of a moving fin with an insulated tip in a convective and radiative environment.

The heat transfer problem to be considered is a one-dimensional (1D) fin moving in a convective-radiative environment, as shown in Fig. 1. The length of the fin is denoted as *L*, its uniform cross-sectional area as *A*, and its circumferential perimeter of the cross-section as *P*, respectively. The fin consists of homogeneous and isotropic material, which as an extended surface completely adheres to a base having given temperature. This situation may occur in metal extrusion, hot rolling processes, etc., where the hot plate from a die or furnace is kept at a constant temperature, $T_{b}$. The heat sink temperatures are supposed to be $T_{a}$ for convection and $T_{s}$ for radiation, respectively. In the present study, the fin material is assumed to continuously move horizontally with a constant speed *U*. Heat loss on the fin surface takes place by convection and radiation. We assume that fin heat lost from its surface radiation satisfies the well-known nonlinear Stefan-Boltzmann law. In the absence of heat source or sink, the 1D version of the governing ordinary differential equation for the above-stated nonlinear steady heat transfer problem for moving fins, therefore, reads [24], [26], [32], [34]

(1) $$\frac{d}{dX}\left(k\left(T\right)A\frac{dT}{dX}\right)-Ph\left(T-T_{a}\right)-\varepsilon \sigma P\left(T^{4}-T_{s}^{4}\right)+\rho c_{p}UA\frac{dT}{dX}=0,\;\;0<X<L$$

where *h* is a constant designating heat convection transfer coefficient, *k* thermal conductivity that is a function depending on the temperature, *ε* surface emissivity, *σ* Stefan-Boltzmann constant, *ρ* the density, $c_{p}$ the specific heat of the material, and *X* the location measured from the fin tip. For simplicity, the temperature at fin's base is assumed unchanged. Also, the temperature in the convection environment remains constant. It is mentioned that an opposite velocity direction, i.e. $U=-U_{0}$, may be applied instead of the above equation, e.g. in [33], [49]. For the latter case, an opposite conclusion can be inferred when one replaces *U* with a negative $-U_{0}$. Usually, the thermal conductivity in the fin is constant for lower temperature change. When the temperature changes large enough, it is no longer uniform. In what follows, the case of thermal conductivity *k* being proportional to the temperature change is considered, namely

(2) $$k\left(T\right)=k_{a}\left(1+\lambda \frac{T-T_{a}}{T_{b}-T_{a}}\right),$$

where $k_{a}$ is the thermal conductivity parameter, $T_{a}$ denotes the surrounding fluid temperature, $T_{b}$ specifies the base temperature, *λ* is a non-dimensional constant. For practical situations, the parameter *λ* must satisfy an additional condition to guarantee the requirement of a positive thermal conductivity. The surface emissivity *ε*, the convection heat transfer coefficient *h*, and the geometric parameters A,P are supposed to be unchanged and moreover they are independent of the temperature. Clearly, the independence of *A* and *P* on the temperature indicates that expansion or shrinkage of the fin due to increase or decrease of temperature along with associated influence is neglected. For a fin with rectangular cross-section, we have A=2tw,P=2(2t+w), where 2*t* and *w* denote the thickness and width of the cross-section. Usually, one may assume that *w* is unchanged, while *t* may vary with the local position. For several typical fin profiles, the thickness *t* may be expressed as $t_{b}+\delta \left[{\left(x/L\right)}^{\beta }-1\right]$, where *β* is usually taken as 0 for rectangular fins, 1 for trapezoidal fins, and larger than 1 for concave fins, respectively (see Fig. 1). It is worth noting that for variable cross-section profiles, one takes A/P=t when t<<w. For convenience, in the following analysis, we assume t<<w and choose *t* as a constant.

In this work, the surrounding environment is opted to be nearly absorbing or black and its temperature takes the same value as the surrounding fluid, viz. $T_{s}=T_{a}$ [24]. Actually, for different values of $T_{s}$ and $T_{a}$, the treatment is similar by a slight modification of the used method. Furthermore, owing to a great jump between *T* and $T_{a}$, we may simply assume $T_{s}=T_{a}=0$, as used in [50]. In addition, in practice, heat loss from the fin tip is low enough as compared to heat loss from the fin surface and is then discarded. Thus, the adiabatic fin tip is assumed and boundary conditions read below

(3) $$\frac{dT}{dX}=0,\;\text{at }X=0,$$

(4) $$T=T_{b},\;\text{at }X=L.$$

It is also mentioned that for fin's tip and fin's base, more general boundary conditions containing both convection and radiation heat transfer can be adopted and analogously analyzed [51].

For convenience of later analysis, similar to the previous studies [9], [34], the following non-dimensional variables are introduced

(5) $$\theta =\frac{T-T_{a}}{T_{b}-T_{a}},\;\;x=\frac{X}{L},\;\;x^{⁎}=lx,\;\;\;l=\frac{LP}{A}=\frac{L}{t},$$

(6) $$\;\;\;\;\alpha =\frac{k_{a}}{\rho c_{p}},\;\;\;Bi=\frac{hA}{k_{a}P},\;\;Bi_{r}=\frac{h_{r}A}{k_{a}P},\;Pe=\frac{UA}{\alpha P},$$

(7) $$N_{c}=\frac{h_{r}L}{k_{a}}\frac{L}{t},\;N_{r}=\frac{h_{r}L}{k_{a}}\frac{L}{t},\;h_{r}=\varepsilon \sigma T_{b}^{3},\;N_{m}=Pe\frac{L}{t}$$

where *θ* is the non-dimensional temperature, *α* thermal diffusivity, *Bi* Biot number, *Pe* Peclet number, and *l* the area ratio of the lateral area to the cross-sectional area. In the above, $h_{r}$ and $Bi_{r}$ are the radiation heat transfer coefficient and the Biot number related to the radiation, respectively. The parameters $N_{c}$ and $N_{r}$ indicate the convection-conduction number and the radiation-conduction number, respectively, which describe the ratio of heat transferred by convection on the fin lateral surface *hLP* to the heat transferred by conduction $k_{a}A/L$, and the ratio of the heat transferred by radiation on the fin lateral surface $h_{r}LP$ to the heat transferred by conduction $k_{a}A/L$. In fact, the characteristic dimension of the fin in the transverse direction may be taken to be A/P; so *l* also indicates the ratio of the fin's length to the characteristic length. For heat flow in the moving fin, Bi<<1 is required and the transverse variation of *T* at an axial position is understood to be uniform over the cross-section. Hence, the problem can be treated as 1D heat transfer model. Since Bi<<1 and enough large *l* are required, in the following the dimensionless parameter $N_{c}$ ranges between 0 and 4, implying from convection-free to strong convection, respectively, while the other dimensionless parameter $N_{r}$ usually lies from 0 to 1.2 [1]. Then, the 1D version of the nonlinear heat transfer balance equation (1) is then rewritten below:

(8) $$\frac{d}{dx^{⁎}}\left[\left(1+\lambda \theta \right)\frac{d\theta }{dx^{⁎}}\right]-Bi\theta -Bi_{r}\theta^{4}+Pe\frac{d\theta }{dx^{⁎}}=0,\;\;0<x^{⁎}<l,$$

or

(9) $$\frac{d}{dx}\left[\left(1+\lambda \theta \right)\frac{d\theta }{dx}\right]-N_{c}\theta -N_{r}\theta^{4}+N_{m}\frac{d\theta }{dx}=0,\;\;0<x<1.$$

When the material or the fin is stationary, i.e. Pe=0, Eq. (8) reduces to

(10) $$\left(1+\lambda \theta \right)\frac{d^{2}\theta }{dx^{⁎2}}+\lambda {\left(\frac{d\theta }{dx^{⁎}}\right)}^{2}-Bi\theta -Bi_{r}\theta^{4}=0,\;\;0<x^{⁎}<l,$$

identical to the nonlinear equation of a stationary fin exposed in convective-radiative environment [16].

In addition, the boundary conditions stated above (3) and (4) are expressed according to the non-dimensional variables:

(11) $$\frac{d\theta }{dx}\left(0\right)=0,\;\;\;\theta \left(1\right)=1.$$

## 3 Approximate temperature distribution

Since Eq. (9) is a nonlinear ordinary differential equation, it is unlikely to obtain explicit exact solutions. In this section, we provide a feasible and convenient approach to derive a simple expression for the temperature field with allowable accuracy. We begin our derivation with Eq. (9) or

(12) $$\frac{d^{2}}{dx^{2}}\left(\theta +\frac{\lambda }{2}\theta^{2}\right)-N_{c}\theta -N_{r}\theta^{4}+N_{m}\frac{d\theta }{dx}=0.$$

For the above-resulting ordinary differential equation (12), one integrates its sides two times with respect to *x*, respectively, and obtains

(13) $$\left(1+\lambda \theta \right)\frac{d\theta }{dx}-\int_{0}^{x}\left[N_{c}\theta \left(s\right)+N_{r}\theta^{4}\left(s\right)\right]ds+N_{m}\theta =C_{1}$$

and

(14) $$\theta \left(x\right)+\frac{\lambda }{2}\theta^{2}\left(x\right)-\int_{0}^{x}\left(x-s\right)\left[N_{c}\theta \left(s\right)+N_{r}\theta^{4}\left(s\right)\right]ds+N_{m}\int_{0}^{x}\theta \left(s\right)ds=C_{1}x+C_{2}$$

where $C_{1}$ and $C_{2}$ are two integration constants to be determined. Clearly, remembering the conditions in (11), by setting x=0 in Eq. (13) and x=1 in Eq. (14), we find

(15) $$N_{m}\theta_{0}=C_{1},$$

(16) $$1+\frac{\lambda }{2}+\int_{0}^{1}\left(s-1\right)\left[N_{c}\theta \left(s\right)+N_{r}\theta^{4}\left(s\right)\right]ds+N_{m}\int_{0}^{1}\theta \left(s\right)ds=C_{1}+C_{2},$$

where $\theta_{0}$ specifies θ(0) at the fin tip. Once $C_{1}$ and $C_{2}$ are determined from Eqs. (15) and (16), we plug them into Eqs. (13) and (14). After eliminating $C_{1}$ and $C_{2}$, from Eq. (14) we obtain a nonlinear integral equation of Fredholm type on *θ* below:

(17) $$\theta \left(x\right)+\frac{\lambda }{2}\theta^{2}\left(x\right)+\int_{0}^{1}R\left(x,s\right)\left[N_{c}\theta \left(s\right)+N_{r}\theta^{4}\left(s\right)\right]ds-N_{m}\int_{x}^{1}\left[\theta \left(s\right)-\theta_{0}\right]ds=1+\frac{\lambda }{2}$$

where the kernel R(x,s) takes 1−x for s<x and 1−s for s>x. It is easily found that the above equation is a standard nonlinear Fredholm equation on the unknown *θ*.

The remaining task is to determine the solution of nonlinear Fredholm Eq. (17). To this end, considering that the insulated fin tip has the boundary condition dθ/dx=0 at the fin tip x=0, we prefer to choose a simple quadratic polynomial to achieve an approximate temperature field rather than a higher-order polynomial by taking more terms to acquire higher accuracy. Hence we assume

(18) $$\theta \left(x\right)=c_{1}+c_{2}x^{2},$$

where $c_{1}$ and $c_{2}$ are unknown constants to be determined.

Now, we denote the residual difference due to the above approximation (18) as

(19) $$\Delta \left(x\right)=\theta \left(x\right)+\frac{\lambda }{2}\theta^{2}\left(x\right)+\int_{0}^{1}R\left(x,s\right)\left[N_{c}\theta \left(s\right)+N_{r}\theta^{4}\left(s\right)\right]ds-N_{m}\int_{x}^{1}\left[\theta \left(s\right)-\theta_{0}\right]ds-\left(1+\frac{\lambda }{2}\right).$$

Clearly, the exact solution is required for the residual difference to vanish everywhere. Since the approximation expression (18) is chosen, it gives rise to Δ(x)≠0. In the following, one therefore may choose the optimal values such that it makes Δ(x) sufficiently tend to zero almost everywhere. To achieve this, we demand

(20) $$\int_{0}^{1}\Delta \left(x\right)dx=0.$$

Next, substituting the expression (18) into Eq. (20) and performing the integrals, after collection we finally get

(21) $$\frac{3+N_{c}}{3}c_{1}+\frac{4\left(5+N_{c}\right)-15N_{m}}{60}c_{2}+\lambda \left(\frac{1}{2}c_{1}^{2}+\frac{1}{10}c_{2}^{2}+\frac{1}{3}c_{1}c_{2}\right)+N_{r}\left(\frac{1}{3}c_{1}^{4}+\frac{4}{15}c_{1}^{3}c_{2}+\frac{6}{35}c_{1}^{2}c_{2}^{2}+\frac{4}{63}c_{1}c_{2}^{3}+\frac{1}{99}c_{2}^{4}\right)=1+\frac{\lambda }{2}.$$

Taking into account the boundary condition θ=1 at x=1, one has

(22) $$c_{1}+c_{2}=1.$$

Eliminating the unknown constant $c_{1}$ from (20) and (22) one gets

(23) $$N_{c}+N_{r}-2c_{2}\left[1+\lambda +\frac{2}{5}N_{m}\left(N_{c}+4N_{r}\right)+\frac{3}{8}N_{m}\right]+\frac{28}{35}\left(\lambda +\frac{36}{7}N_{r}\right)c_{2}^{2}-\frac{256}{105}N_{r}c_{2}^{3}+\frac{128}{231}N_{r}c_{2}^{4}=0.$$

This is a quartic equation in $c_{2}$. Despite the formula available determining the exact roots of a quartic equation, to avert an extremely complicated and burdensome expression for the exact root to Eq. (23), we prefer giving an approximate simple solution to obtaining the exact root to Eq. (23). We then give an approximate root in a relatively simple form

(24) $$c_{2}=\frac{N_{c}+N_{r}}{2\left[1+\lambda +0.4N_{m}\left(N_{c}+4N_{r}\right)+0.375N_{m}\right]}\left(1+\frac{0.2\left(\lambda +5.14N_{r}\right)\left(N_{c}+N_{r}\right)}{{\left[1+\lambda +0.4N_{m}\left(N_{c}+4N_{r}\right)+0.375N_{m}\right]}^{2}}\right).$$

With the above $c_{2}$, the dimensionless temperature distribution in the straight fin can be obtained. In particular, it may be given by the following simple quadratic polynomial

(25) $$\theta \left(x\right)=1-\frac{N_{c}+N_{r}}{2\left[1+\lambda +0.4N_{m}\left(N_{c}+4N_{r}\right)+0.375N_{m}\right]}\left(1+\frac{0.2\left(\lambda +5.14N_{r}\right)\left(N_{c}+N_{r}\right)}{{\left[1+\lambda +0.4N_{m}\left(N_{c}+4N_{r}\right)+0.375N_{m}\right]}^{2}}\right)\left(1-x^{2}\right).$$

It can be utilized to estimate the temperature distribution along the fin and its effectiveness will be demonstrated subsequently. When the above temperature change is prescribed, the tip temperature of the fin and the temperature gradient are written below, respectively

(26) $$\theta_{0}=1-\frac{N_{c}+N_{r}}{2\left[1+\lambda +0.4N_{m}\left(N_{c}+4N_{r}\right)+0.375N_{m}\right]}\left(1+\frac{0.2\left(\lambda +5.14N_{r}\right)\left(N_{c}+N_{r}\right)}{{\left[1+\lambda +0.4N_{m}\left(N_{c}+4N_{r}\right)+0.375N_{m}\right]}^{2}}\right),$$

(27) $$\frac{d\theta }{dx}=\frac{x\left(N_{c}+N_{r}\right)}{1+\lambda +0.4N_{m}\left(N_{c}+4N_{r}\right)+0.375N_{m}}\left(1+\frac{0.2\left(\lambda +5.14N_{r}\right)\left(N_{c}+N_{r}\right)}{{\left[1+\lambda +0.4N_{m}\left(N_{c}+4N_{r}\right)+0.375N_{m}\right]}^{2}}\right).$$

From the above, we find that the obtained results give an explicit expression for approximately estimating the temperature and the temperature gradient. Its accuracy will be examined in what follows. In particular, these simple relationships can reflect a definite analytic dependence relationship of the temperature and the temperature gradient on the heat properties or relevant parameters. Usually, the other numerical method fail to give such simple relationships, notwithstanding the variation of the temperature (gradient) versus the heat parameters can be plotted from the numerical results.

## 4 Fin efficiency

In assessing the thermal performance of fins, there are significant parameters such as the fin efficiency, the fin effectiveness, the fin resistance, etc [52]. Here, as a representative, we examine the dependence of the fin efficiency on the non-dimensional numbers $N_{c},N_{r}$, and $N_{m}$. According to the definition of the fin efficiency, it is defined as the ratio of the actual heat loss at the surface to the total heat if the whole fin has the same base temperature, namely

(28) $$\eta =\frac{Q_{actural}}{Q_{ideal}}$$

where the actual heat loss is composed of three parts

(29) $$Q_{actual}=Q_{c}+Q_{r}+Q_{m},$$

with

(30) $$Q_{c}=\int_{0}^{L}hP\left(T-T_{a}\right)dX,\;\;Q_{r}=\int_{0}^{L}\varepsilon \sigma P\left(T^{4}-T_{s}^{4}\right)dX,\;\;Q_{m}=-\rho c_{p}UA\left(T_{b}-T_{0}\right).$$

If the fin surface takes the base temperature, the ideal heat loss is

(31) $$Q_{ideal}=hPL\left(T_{b}-T_{a}\right)+\varepsilon \sigma PL\left(T_{b}^{4}-T_{s}^{4}\right)-\rho c_{p}UA\left(T_{b}-T_{0}\right).$$

Because of the energy conversation, under the assumption of $T_{a}=T_{s}=0$, we easily find

(32) $$\eta =\frac{\int_{0}^{L}hP\left(T-T_{a}\right)dX+\int_{0}^{L}\varepsilon \sigma P\left(T^{4}-T_{s}^{4}\right)dX-\rho c_{p}UA\left(T_{b}-T_{0}\right)}{hPL\left(T_{b}-T_{a}\right)+\varepsilon \sigma PL\left(T_{b}^{4}-T_{s}^{4}\right)-\rho c_{p}UA\left(T_{b}-T_{0}\right)}=\frac{N_{c}\int_{0}^{1}\theta \left(x\right)dx+N_{r}\int_{0}^{1}\theta^{4}\left(x\right)dx-N_{m}\left(1-\theta_{0}\right)}{N_{c}+N_{r}-N_{m}\left(1-\theta_{0}\right)}.$$

The above result provides an approach to acquire the fin efficiency through the tip temperature together with the whole temperature. In the above expression for assessing the fin efficiency, the heat losses from convection, radiation, and motion are all considered. It is mentioned that in the previous studies, the heat loss due to the fin movement is often neglected such as [8], [30], [32], [37], [39], [46]. This effect has been taken into account in [34], [49]. It is mentioned that an alternative approach for determining the fin efficiency is based on the tip temperature and the temperature gradient at the base, i.e.

(33) $$\eta =\frac{\left(1+\lambda \right)\theta^{\prime }\left(1\right)}{N_{c}+N_{r}-N_{m}\left(1-\theta_{0}\right)}.$$

If $\theta^{\prime }\left(1\right)$ is exactly given, the above result provides a simple approach to determining the fin efficiency. For example, when $N_{m}=0$ or $N_{r}=0$, some researchers indeed applied this expression to evaluate *η* e.g. [8], [35]. Nevertheless, since our solution is approximate, not the exact one, taking account of the fact that the sensitivity of the derivative is much larger than that of the integral, we prefer to select (32) to evaluate the fin efficiency rather than (33). Thus, using the above-derived approximate non-dimensional temperature distribution, the fin efficiency can be expressed by

(34) $$\eta =1-\frac{2\left(N_{c}+4N_{r}\right)}{3\left(N_{c}+N_{r}-N_{m}c_{2}\right)}c_{2}+\frac{16N_{r}}{5\left(N_{c}+N_{r}-N_{m}c_{2}\right)}\left(\frac{8}{63}c_{2}^{2}-\frac{4}{7}c_{2}+1\right)c_{2}^{2},$$

where $c_{2}$ is provided by (24).

In closing this section, we conclude that the present method provides a feasible way for getting a simple expression for the temperature distribution and the fin efficiency. In particular, the derived relationship is analytic, rather than numerical. In other words, with the help of the derived results, one can further design fins to achieve the desired purpose. Moreover, this approach is unified and may be extended to apply other cases including the heat loss of porous fins with mass flow.

## 5 Results and discussion

In the foregoing, we have obtained the approximate temperature distribution along the fin length. Prior to the presentation of numerical results, let us consider some special cases of (34). For a stationary fin with constant thermal conductivity, meaning $N_{m}=0$, the previous result derived in [15] is recovered from the above. Furthermore, if in the absence of radiation loss, we have $\lambda =N_{m}=N_{r}=0$, the above-obtained approximation of the temperature excess simplifies to

(35) $$\theta \left(x\right)=1-\frac{N_{c}}{2\left(1+0.4N_{c}\right)}\left(1-x^{2}\right),$$

and the fin efficiency becomes $\eta =1/\left(1+0.4N_{c}\right)$, identical to those derived by Sobamowo [53].

To verify the effectiveness of the suggested approximation method of the temperature excess in connection with the fin efficiency, we make a comparison of the accuracy between the obtained numerical results and the available previous results in Table 1. In the following calculations, l=1 is presumed for comparison. Table 1 gave a comparison of our approximate results of the non-dimensional fin tip temperature $\theta_{0}$ for a moving fin with typical values of the non-dimensional numbers. In previous studies, the differentiation transformation method [26] and the Adomian decomposition method [30] were applied to calculate the numerical results, which are also tabulated in Table 1 for comparison. It is seen that our approximate results agree well with the exact ones and those given in [26], [30]. If denoting the maximum relative error

(36) $$error\text{\%}=\max100\left|\frac{present-previous}{present}\right|$$

we also list the maximum relative errors in Table 1 and find that the maximum relative error does not exceed 1.3%. Moreover, these maximum relative errors occur when comparing our results with those based on the Adomian decomposition method at λ=0.75 [30]. In particular, the values of the tip temperature $\theta_{0}$ for λ=1 were not given in [30]. It implies that the results according to the Adomian decomposition method possibly deteriorate and become more inaccurate when *λ* becomes larger. However, the comparison also shows that our results have a very satisfactory agreement with those based on the differentiation transformation method [26] and the maximum relative error is less than 0.3%. On the other hand, irrespective of using either the Adomian decomposition method or the differentiation transformation method or others else such as the homotopy analysis method [25], the form of their solutions is quite complicated. Explicit analytical expressions were never reported for the temperature excess and the fin efficiency, although numerical results were given. It is worth pointing out that there are apparent advantages if explicit expressions are provided, while the numerical results are inconvenient for practical engineering applications. It should be mentioned that Bouaziz and Aziz [50] used the double optimal linearization method to give an explicit solution with a relatively simple expression for a stationary nonlinear fin problem with convection and radiation environments. As compared to the above results, the explicit expression (25) provides a both simpler and enough accurate expression for not only the temperature excess (as well as the temperature gradient) but also the fin efficiency with high accuracy with the maximum relative error less than 1.26% for the range of the data in Table 1. From Table 1, it is seen that when $N_{r},N_{c}$ and *Pe* become larger and larger, the error between our results and those numerical results based on the Adomian decomposition method and the differentiation transformation method gradually becomes some large. So, for usual values, e.g. those listed in Table 1, the approximate results have very high accuracy. Nonetheless, for very large $N_{r},N_{c}$ and *Pe* values, one must be cautious in applying this approximate solution. It is interesting to note that our approximation expression is quite simple as compared to those provided by the other approaches [26], [30].

**Table 1:** Comparison of the non-dimensional tip temperature *θ*_0_ for a stationary fin.

							*N*_*r*_			
					0.25				0.5	
*N*_*c*_	Pe	*λ*		Present	DTM [26]	ADM [30]		Present	DTM [26]	ADM [30]
0.25	0.25	0		0.8352	0.8358	0.8358		0.7937	0.7944	0.7944
		0.25		0.8583	0.8584	0.8584		0.8188	0.8190	0.8190
		0.5		0.8757	0.8756	0.8765		0.8385	0.8384	0.8396
		0.75		0.8894	0.8892	0.8902		0.8543	0.8541	0.8595
		1		0.9003	0.9001	-		0.8673	0.8670	-

				max error (%)	0.07%	0.09%			0.08%	0.61%

	0.5	0		0.8452	0.8447	0.8447		0.8045	0.8040	0.8039
		0.25		0.8657	0.8650	0.8651		0.8271	0.8246	0.8265
		0.5		0.8814	0.8808	0.8811		0.8451	0.8443	0.8456
		0.75		0.8939	0.8933	0.8898		0.8597	0.8589	0.8620
		1		0.9040	0.9034	-		0.8717	0.8710	-

				max error (%)	0.08%	0.46%			0.30%	0.27%

0.5	0.25	0		0.7637	0.7652	0.7652		0.7332	0.7340	0.7340
		0.25		0.7953	0.7955	0.7956		0.7648	0.7645	0.7646
		0.5		0.8195	0.8192	0.8215		0.7896	0.7890	0.7908
		0.75		0.8387	0.8381	0.8481		0.8097	0.8089	0.8200
		1		0.8542	0.8536	-		0.8263	0.8255	-

				max error (%)	0.20%	1.11%			0.11%	1.26%

	0.5	0		0.7775	0.7774	0.7774		0.7469	0.7461	0.7461
		0.25		0.8057	0.8049	0.8052		0.7754	0.7741	0.7743
		0.5		0.8277	0.8266	0.8294		0.7981	0.7967	0.7994
		0.75		0.8452	0.8441	0.8523		0.8167	0.8153	0.8271
		1		0.8595	0.8585	-		0.8321	0.8308	-

				max error (%)	0.13%	0.83%			0.18%	1.26%

**Figure 2:**
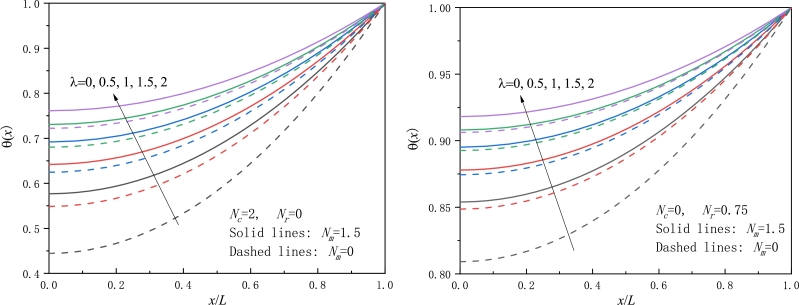
The nondimensional temperature excess for a moving fin with various *λ* values, a) in a pure convective ambience, b) in a pure radiative ambience.

**Figure 3:**
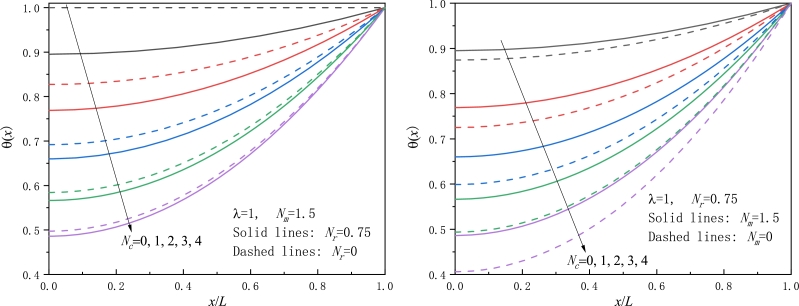
Comparison of the nondimensional temperature excess of a conductive-convective fin, a) with/without radiation loss, b) moving/stationary fin.

**Figure 4:**
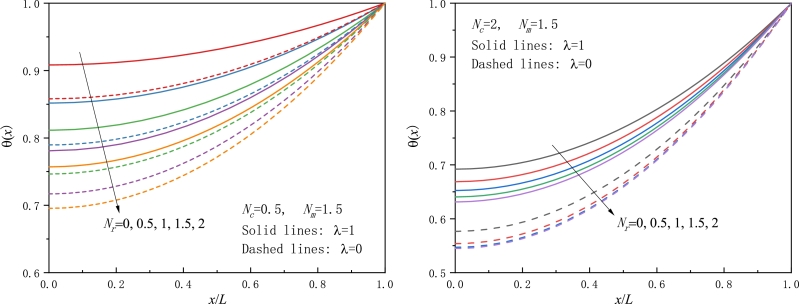
Effect of the radiation parameter *N*_*r*_ on the temperature excess for a moving fin in a convective-radiative ambience, a) *N*_*c*_ = 0.5, b) *N*_*c*_ = 2.

**Figure 5:**
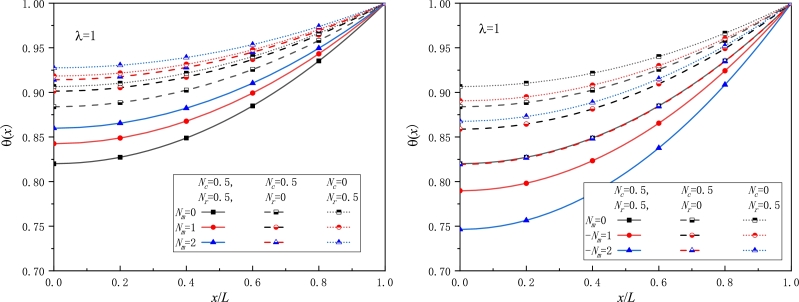
Effect of the movement speed or the Peclet number on the temperature distribution for a moving conductive-convective-radiative fin with a) positive movement speed, b) negative movement speed.

For various values of the non-dimensional parameters, the temperature distribution *θ* is shown in Figure 2, Figure 3, Figure 4, Figure 5. In calculating numerical results, only one non-dimensional parameter is allowed to vary and the other non-dimensional parameters are kept unchanged. The following values of the non-dimensional parameters are used unless otherwise stated: $\lambda =1,\;N_{c}=2,\;N_{r}=0.75,\;N_{m}=1.5$.

Figs. 2a and 2b show the variation of the temperature distribution against the dimensional fin length with λ=0,0.5,1,1.5,2. From Fig. 2a and 2b, it is seen that the local temperature at position *x* rises with the *λ* value becoming larger. It is easily understood since the thermal conductivity is raised, leading to that heat transfers faster along the fin, and then the fin tip has a larger temperature, as observed in Fig. 2a and 2b. This trend is also consistent with that observed in [25], [26]. Moreover, from Fig. 2a and 2b, when neglecting radiation heat loss, the nondimensional temperature somewhat increases as compared to that when radiation heat loss is considered. Fig. 2b also provides a comparison of the nondimensional temperature with different *λ* values for stationary and moving fins.

Figs. 3a and 3b display the effect of the parameter $N_{c}$ on the temperature field where various values of $N_{c}=0,1,2,3,4$ are chosen. Opposite to the effect of the parameter *λ*, an increase in $N_{c}$ leads to a decrease in the temperature change. The reason for that is that when $N_{c}$ becomes large, for a given fin the Biot number *Bi* also becomes greater and then convection heat transfer easily occurs. Thus, the fin surface has much more heat loss, so the temperature excess and the fin tip temperature for large $N_{c}$ or *Bi* values are less than those for lower $N_{c}$ or *Bi* values. This conclusion is in agreement with that in [26], [33], [50]. In particular, in the case of $N_{c}=N_{r}=0$, although the other parameters are non-vanishing, we still find the nondimensional temperature has no response and the whole temperature taking unity, as seen in Fig. 3a, since no heat loss takes place and all heat flowing from the fin base can reach the fin tip. For other cases with either of $N_{c}$ and $N_{r}$ being not zero, the nondimensional temperature gradually decreases with the distance away from the fin tip. Moreover, the larger the parameter $N_{c}$, the lower the temperature change *θ*.

The impact of the radiation parameter $N_{r}$ on the temperature excess is examined for a conductive-radiative-convective fin with $N_{c}=0.5,2$ for Figs. 4a and 4b, respectively. Obviously, for a smaller $N_{c}$ value, i.e. lower heat loss occurs on the fin surface, it is viewed by inspecting Fig. 4a and 4b that the temperature on the whole fin surface is larger than that for a larger $N_{c}$ value, as expected. The same reason holds for radiation heat dissipation from the fin surface. Therefore, for a fin in a convective and radiative environment, there is a notable declination in the temperature for larger values of both $N_{c}$ and $N_{r}$. In other words, both convection and radiation play a crucial role in heat dissipation.

Figs. 5a and 5b are devoted to the examination of the effect of the movement speed or the Peclet number on the temperature distribution. When the fin material moves towards a cooler ambience, the nondimensional speed parameter $N_{m}$ or the Peclet number *Pe* takes a positive value, and the temperature is found to progressively increase with $N_{m}$ rising. This trend is the same as that observed for a fin either in a pure convective environment or in a pure radiative environment in Figs. 2a and 2b and also in [24], [26]. As pointed out before, as the movement direction is opposite, a negative $N_{m}$ is replaced by $N_{m}$. Thus, from Fig. 5b, we find that the larger the movement velocity, the less the temperature field, which is in line with the obtained results in [31], [33], [49]. It is worth noting that one must be particularly cautious in treating the move direction in connection with the coordinate *x* since there are usually two choices on the coordinate of the fin tip being either x=0 or x=L.

**Figure 6:**
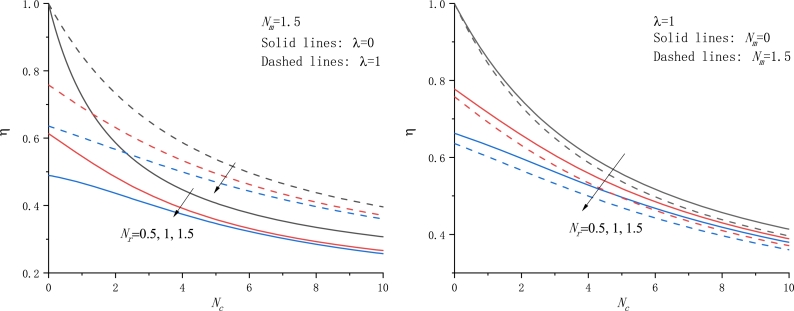
The fin efficiency against the parameter *N*_*c*_ for various values of *N*_*r*_,*N*_*m*_, and *λ*, a) *N*_*m*_ = 1.5, b) *λ* = 1.

**Figure 7:**
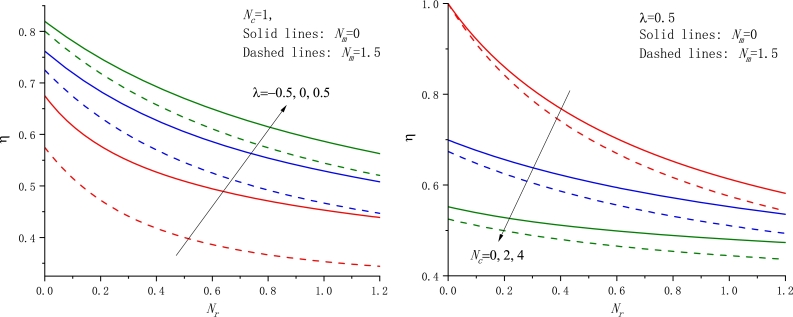
The fin efficiency against the parameter *N*_*r*_ for various values of *N*_*c*_,*N*_*m*_, and *λ*, a) *N*_*c*_ = 1, b) *λ* = 0.5.

**Figure 8:**
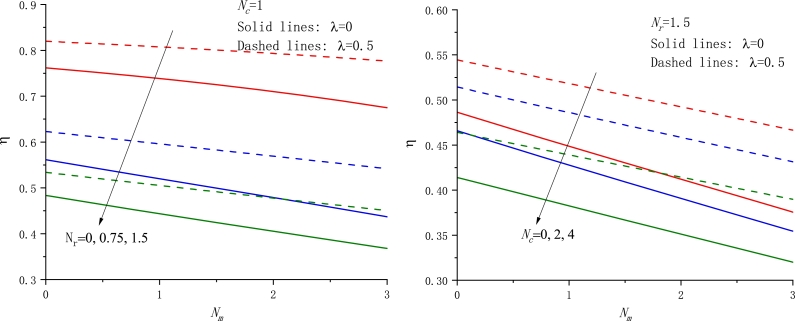
The fin efficiency against the parameter *N*_*m*_ for various values of *N*_*c*_,*N*_*r*_, and *λ*, a) *N*_*c*_ = 1.5, b) *N*_*r*_ = 1.5.

To assess the thermal performance of fins, some parameters such as the fin efficiency, the fin effectiveness, the fin tip temperature, etc. are particularly interesting. In the following, we plot the fin efficiency *η* as a function of the parameter $N_{c}$ with different cases in Figs. 6a and 6b. It is readily found that the fin efficiency always monotonically decreases with $N_{c}$ and $N_{r}$ rising. In addition, the fin efficiency against the other parameters $N_{r}$ and $N_{m}$ is also displayed in Figs. 7a-b and 8a-b, respectively. In particular, the dependence of *η* on $N_{m}$ exhibits an almost linear relationship.

From the above result, the motion of the material from the fin base to a colder ambience gives rise to an increase of the fin efficiency *η* if the other parameters are retained unchanged. This trend is in agreement with the previous conclusion [24], [34]. Of course, if taking a negative motion direction, i.e. $U_{0}=-U$, the opposite conclusion can be drawn. That is, the motion of the material toward the fin base from a colder ambience gives rise to a decrease in the fin efficiency *η* if the other parameters are retained unchanged, in consistency with those [31], [33], [49].

## 6 Conclusions

In this article, a nonlinear heat transfer problem of a moving fin in a convective and radiative environment was studied. Heat loss from surface to the surrounding fluid due to convection and radiation were analyzed. Multi nonlinearities involving temperature-dependent thermal conductivity and heat radiation were considered. A nonlinear heat transfer problem was converted to a nonlinear Fredholm equation that has an approximate quadratic solution. The temperature excess and the fin efficiency were obtained explicitly and analytically, and they possess very simple expressions. The influence of the involved parameters including thermal conductivity, heat convection, radiation, and moving speed on the temperature excess and the fin efficiency were clarified. The obtained explicit solution is helpful in design and optimization. In addition, the present method is easy to extend to treat a moving porous fin with various profiles and mass flow in the fin exposed in convective and radiative environment.

## Funding

This work was supported by the Scientific Research Fund of Hunan Provincial Education Department under Grant No. 24A0229.

## CRediT authorship contribution statement

**Yanqun Han:** Writing – original draft, Validation, Investigation. **Xulong Peng:** Writing – review & editing, Validation, Investigation.

## Declaration of Competing Interest

The authors declare that they have no known competing financial interests or personal relationships that could have appeared to influence the work reported in this paper.
